# Serum Catestatin Level as a Novel Biomarker of Oral Lichen Planus

**DOI:** 10.3390/medsci14030417

**Published:** 2026-07-22

**Authors:** Mia Roglic Karan, Dinko Martinovic, Ema Puizina, Lovre Martinovic, Jasna Puizina, Daniela Supe-Domic, Roko Santic, Josko Bozic, Livia Sukanec

**Affiliations:** 1 Department of Dental Medicine, Primary Healthcare Center of Split-Dalmatia County, 22320 Drnis, Croatia; 2 Department of Maxillofacial Surgery, University Hospital of Split, 21000 Split, Croatia; 3 Department of Maxillofacial Surgery, University of Split School of Medicine, 21000 Split, Croatia; 4 Medical Studies, University of Split School of Medicine, 21000 Split, Croatia; 5 Department of Biology, Faculty of Science, University of Split, 21000 Split, Croatia; 6 Department of Medical Laboratory Diagnostics, Faculty of Health Sciences, University of Split, 21000 Split, Croatia; 7 Department of Medical Laboratory Diagnostics, University Hospital of Split, 21000 Split, Croatia; 8 Department of Pathophysiology, University of Split School of Medicine, 21000 Split, Croatia; 9 Department of Oral Pathology, University of Split School of Medicine, 21000 Split, Croatia; 10 Department of Dental Medicine, University Hospital of Split, 21000 Split, Croatia

**Keywords:** oral lichen planus, catestatin, biomarker, immunomodulation

## Abstract

**Background/Objectives:** Oral lichen planus (OLP) is a chronic immune-mediated disease of the oral mucosa in which T-cell infiltration, epithelial injury, and stress-related neuroendocrine signaling appear to intersect. Catestatin (CST), a peptide generated from chromogranin A (CgA), modulates catecholamine release and inflammatory cell responses; however, its association with localized mucosal inflammation in OLP has not been clarified. This study aimed to compare serum CST levels between OLP patients and healthy controls and to examine their association with clinical subtype, disease severity, and vitamin D status. **Methods:** In this cross-sectional study, 51 patients with clinically and histopathologically confirmed OLP and 60 healthy controls were enrolled at the University Hospital of Split. Serum CST was quantified using ELISA. OLP severity was assessed with the REU score by a single experienced oral medicine examiner who was blinded to laboratory results, and pain and burning were recorded separately using 0–10 VASs. **Results:** Serum CST levels were significantly higher in OLP patients compared to healthy controls (*p* < 0.001). CST levels showed a strong positive correlation with the REU total score (r = 0.781, *p* < 0.001), as well as with VAS pain (r = 0.708, *p* < 0.001) and VAS burning (r = 0.729, *p* < 0.001). Erosive OLP exhibited significantly higher CST levels compared to the non-erosive subtype (*p* < 0.001). Furthermore, serum vitamin D levels were significantly lower in OLP patients (*p* < 0.001), with no significant correlation observed between CST and vitamin D levels. **Conclusions:** Serum CST levels were higher in OLP patients and showed close associations with REU scores and symptoms, with the strongest signal observed in erosive disease. CST may therefore contribute to the neuroendocrine-immune profile of OLP and may be useful as an adjunctive marker of clinically active or erosive disease. Larger prospective studies including salivary and tissue-based CST measurements are needed before CST can be used for routine monitoring.

## 1. Introduction

Oral lichen planus (OLP) is a chronic immune-mediated disorder of the oral mucosa, estimated to affect roughly 0.5% to 2% of the population and observed most often in middle-aged women [1]. Clinically, OLP can appear as reticular, atrophic, erosive, plaque-like, papular or bullous lesions, with relapsing and remitting symptoms that often require long-term follow-up [2]. Its etiology has not been fully resolved, but epithelial injury is considered to be driven primarily by cytotoxic T-cell responses, together with genetic susceptibility, psychological stress, viral triggers, and systemic autoimmune influences [3]. Because OLP is classified by the World Health Organization as an oral potentially malignant disorder, surveillance remains important; published malignant transformation estimates approach 1.5% in some series [4].

Catestatin (CST) is a 21-amino-acid peptide (hCgA352-372) produced by proteolytic processing of chromogranin A (CgA), a secretory granule protein released with catecholamines from adrenal chromaffin cells and other neuroendocrine tissues [5]. Originally described as an inhibitor of nicotinic cholinergic catecholamine release, CST is now recognized as a multifunctional peptide involved in cardiovascular regulation, antimicrobial defense, metabolic control, and innate immune signaling [6,7,8]. In inflammation, CST promotes a shift from classically activated M1 macrophages toward an M2-like phenotype, decreases TNF-alpha, IL-1beta and IL-6 production, restrains leukocyte recruitment, and down-regulates endothelial adhesion molecules [9,10]. Altered circulating CST concentrations have been described in rheumatoid arthritis, inflammatory bowel disease, ankylosing spondylitis and COVID-19, supporting its relevance as a systemic marker of inflammatory activity [5,6,7,8,10].

Although OLP manifests locally in the oral mucosa, its inflammatory microenvironment is influenced by systemic neuroendocrine and immune inputs. Psychological stress, a commonly reported disease modifier, activates the hypothalamic–pituitary–adrenal axis and the sympathoadrenal system, which promote catecholamine and CgA release [11,12]. Because CgA is the precursor of CST, changes in sympathoadrenal signaling could alter CST availability in parallel with OLP activity. More importantly, systemic CST can interact with pathways that are directly relevant to oral mucosal inflammation, including endothelial adhesion molecule expression, chemokine-driven leukocyte trafficking, macrophage polarization, and T-cell-dominated epithelial damage [10,13,14,15]. These mechanisms provide a biologically plausible bridge between a circulating neuroendocrine-derived peptide and the local homing and activation of lymphocytes in OLP lesions. Previous studies have examined biomarkers such as angiopoietin-2, vitamin D, immunoglobulins and salivary cortisol in OLP, but serum catestatin has not yet been specifically evaluated [16,17].

Accordingly, this study measured serum CST levels in patients with OLP and healthy controls. We also examined whether CST concentrations differ by erosive status and whether they correlate with REU-based disease activity, symptom intensity, and vitamin D concentrations.

## 2. Materials and Methods

### 2.1. Study Design and Ethical Considerations

This cross-sectional study was carried out at the Department of Oral Pathology, University Hospital of Split, between April 2023 and November 2025.

The protocol was approved by the Ethics Committee of the University Hospital of Split (No. 2181-147/01/06/LJ.Z.-23-03; 14 March 2023). The study followed the Declaration of Helsinki, and all participants received study information before providing written informed consent.

### 2.2. Subjects

Patients with OLP were recruited from the Department of Oral Pathology, University Hospital of Split. Participation was voluntary and anonymized, and all patients received standard clinical care. Seventy-three patients were screened; 22 were excluded because at least one inclusion or exclusion criterion was not met. Medication history was recorded at enrollment, and blood was drawn before any new OLP-directed topical or systemic treatment was initiated.

Controls were healthy volunteers attending routine dental/oral check-ups at the same institution. They had no oral mucosal lesions and no history or clinical evidence of oral inflammatory, autoimmune, potentially malignant, or malignant disease. The same exclusion criteria applied to controls and OLP patients. Current smoking was defined as any active cigarette use within the previous 30 days. Everyday alcohol consumption was recorded as daily intake of alcoholic beverages.

Inclusion criteria were age 18–70 years and a diagnosis of OLP.

Exclusion criteria were active malignancy; chronic renal or hepatic disease; cardiovascular disease, including arterial hypertension and use of antihypertensive medication; diabetes mellitus; rheumatologic or autoimmune disease; oral vitamin D supplementation; psychoactive substance abuse; and current topical or systemic corticosteroid therapy or other OLP-directed immunomodulatory treatment at the time of sampling.

### 2.3. Laboratory and Clinical Examination

All participants underwent medical history review, physical examination, anthropometric measurements, and laboratory testing. OLP diagnosis was established using clinical and histopathological criteria according to the modified World Health Organization diagnostic framework summarized in recent diagnostic recommendations [3]. Potential controls with clinical or laboratory evidence of inflammation were excluded; C-reactive protein and leukocyte count were used as laboratory screening markers. Body height and weight were measured with a calibrated medical scale with an integrated stadiometer (Seca, Birmingham, UK), and BMI was calculated as weight in kilograms divided by height in meters squared.

After a 12-h overnight fast, venous blood was collected from the cubital vein. Routine laboratory testing was performed on the day of sampling, while serum aliquots intended for CST analysis were centrifuged and stored at −80 °C until assay. All analyses followed standard laboratory procedures and were performed by the same experienced biochemist, who was blinded to group allocation.

Serum CST concentrations were measured by enzyme-linked immunosorbent assay using a commercially available kit (EK-053-27CE EIA kit, Phoenix Pharmaceuticals Inc., Burlingame, CA, USA). The manufacturer’s stated assay sensitivity was 0.05 ng/mL, with a measurement range of 0.05–100 ng/mL. Intra-assay and inter-assay coefficients of variation were <10% and <15%, respectively. Because no universally accepted clinical reference interval for serum CST is available for this assay, CST values were analyzed as continuous variables and compared between study groups.

Serum 25-hydroxyvitamin D [25(OH)D] was quantified using Elecsys Vitamin D (Roche Diagnostics International Ltd., Rotkreuz, Switzerland), an electrochemiluminescence immunoassay (ECLIA). Results were expressed in nmol/L, and the analytical measuring range was 7.5–250 nmol/L. For interpretation, vitamin D deficiency was considered <50 nmol/L, insufficiency 50–74.9 nmol/L, and sufficiency ≥ 75 nmol/L; however, the present analysis used continuous vitamin D values because individual category counts were not available for all analyses.

### 2.4. Assessment of Disease Activity

Clinical activity was graded with the Reticulation/Keratosis, Erythema, Ulceration (REU) system, a semi-quantitative clinician-reported tool that scores OLP lesions at ten intraoral sites [18]. A single experienced oral medicine specialist performed all REU assessments while blinded to CST and vitamin D results, using a standardized scoring sheet. Because only one examiner carried out the clinical grading and no repeat-scoring session was built into the protocol, formal inter-examiner and intra-examiner reliability coefficients were not available. At each site, reticulation/keratosis was scored as absent/present, erythema was scored from 0 to 3 according to surface area, and ulceration was scored from 0 to 3 according to ulcer size. The total REU score was calculated as (R × 1) + (E × 2) + (U × 3).

Patients were classified as erosive if at least one erosive/ulcerative lesion was present at any oral site, regardless of concomitant reticular, papular, plaque-like, atrophic, or bullous changes. The non-erosive group included patients without erosive/ulcerative lesions; this group could include reticular, papular, plaque-like, or atrophic lesions.

Pain and burning were recorded separately on two distinct 0–10 visual analog scales (VAS). Patients were asked to rate their average spontaneous symptoms during the preceding 7 days, rather than provoked symptoms during eating, swallowing, or toothbrushing. A score of 0 represented no pain/burning, and 10 represented the worst imaginable pain/burning.

### 2.5. Sample Size Analysis

Sample size was estimated from pilot data obtained in 10 OLP patients and 10 matched controls. Serum CST, the primary outcome, had a mean concentration of 10.42 ± 5.75 ng/mL in the OLP group and 5.11 ± 3.11 ng/mL in controls. Assuming type I error of 0.05 and 90% power, at least 17 participants were required in each group.

### 2.6. Statistical Analyses

Statistical analyses were performed in MedCalc (MedCalc Software, Ostend, Belgium, version 23.5.5). Continuous variables are shown as mean ± standard deviation or median with interquartile range, depending on distribution, while categorical variables are reported as *N* (%). Normality was assessed with the Kolmogorov–Smirnov test. Normally distributed variables were compared using the independent-samples *t*-test; non-normally distributed variables were analyzed with the Mann–Whitney U test. Categorical variables were compared using the chi-square test or Fisher’s exact test when appropriate. Associations between CST and clinical variables were tested with Pearson’s or Spearman’s correlation coefficients according to distribution. Statistical significance was set at *p* < 0.05.

## 3. Results

Overall, 111 participants were analyzed: 60 controls and 51 patients with OLP (Table 1). The groups did not differ significantly in age (*p* = 0.331), BMI (*p* = 0.479), sex (*p* = 0.197), smoking status (*p* = 0.826), or everyday alcohol consumption (*p* = 0.405). Unless otherwise indicated, values in parentheses represent either percentages for categorical variables or interquartile ranges for skewed continuous variables.

Reticular OLP was the most common clinical pattern (47/51, 92.2%), whereas papular, atrophic and bullous types were each recorded in one patient (2.0%) (Table 2). Because multiple lesion types and sites could coexist, erosive/ulcerative lesions were present in 35 patients (68.6%), and 16 patients (31.4%) were classified as non-erosive. The buccal mucosa was the most frequent site (92.2%), while the oral floor was least often involved (5.9%).

**Figure 1:**
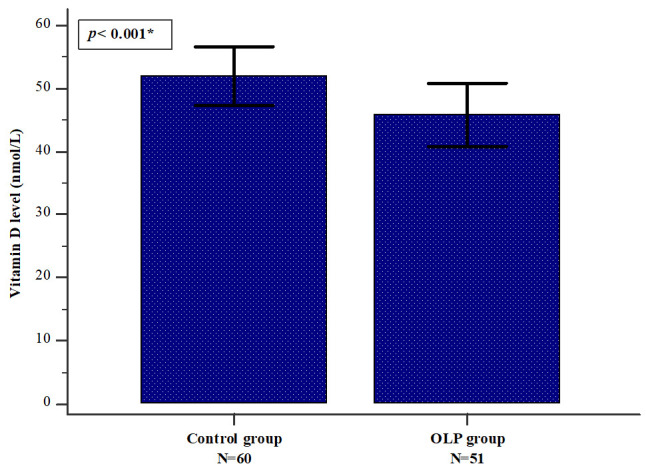
Comparison of vitamin D levels between the control and OLP group. All variables are presented as mean ± standard deviation. * student *t*-test.

Serum vitamin D concentrations were lower in the OLP group than in controls (45.1 ± 17.8 vs. 53.4 ± 18.07 nmol/L, *p* < 0.001) (Figure 1). Based on commonly used thresholds, both mean values fall below the sufficiency cut-off of 75 nmol/L.

**Figure 2:**
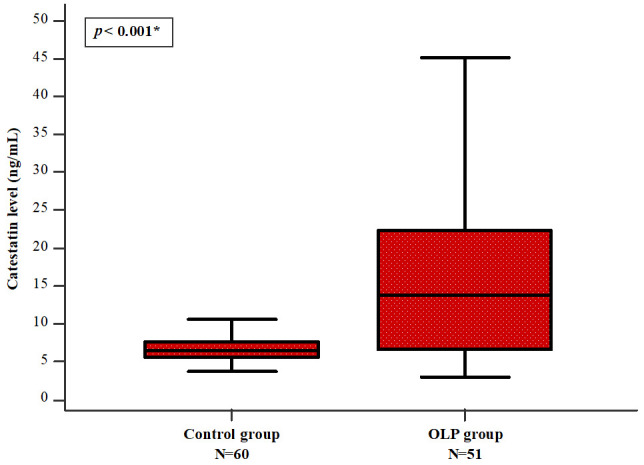
Comparison of catestatin serum levels between the control group and the OLP group. All variables are presented as median (interquartile range). * Mann–Whitney U test.

Median serum CST was higher in patients with OLP than in controls (13.8 [6.9–25.0] vs. 6.4 [5.5–7.6] ng/mL, *p* < 0.001) (Figure 2).

Within the OLP group, serum CST showed strong positive correlations with REU-E score (r = 0.748, *p* < 0.001), REU-U score (r = 0.770, *p* < 0.001), REU total score (r = 0.781, *p* < 0.001), VAS pain (r = 0.708, *p* < 0.001), and VAS burning (r = 0.729, *p* < 0.001) (Table 3). No other statistically significant correlations were observed.

**Figure 3:**
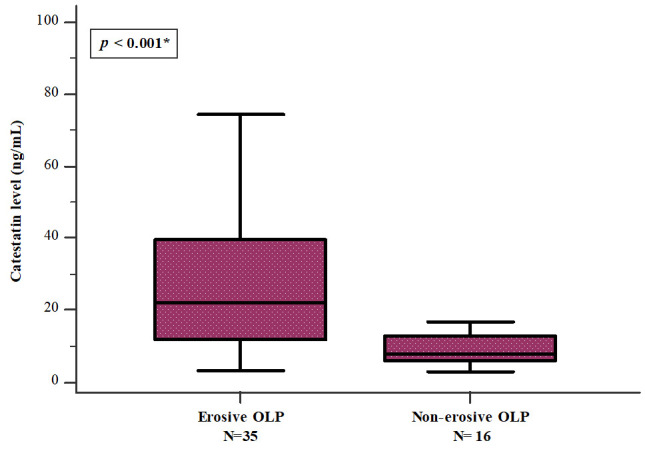
Comparison of serum catestatin levels between the non-erosive and erosive OLP form groups. All variables are presented as median (interquartile range). * Mann–Whitney U test.

Exploratory subtype analysis showed higher serum CST in erosive OLP than in non-erosive OLP (21.9 [11.8–39.4] vs. 7.7 [5.7–12.7] ng/mL, *p* < 0.001) (Figure 3). Descriptively, the non-erosive OLP median was close to the control median (7.7 vs. 6.4 ng/mL), suggesting that the overall OLP-control difference was mainly driven by the erosive subgroup.

**Figure 4:**
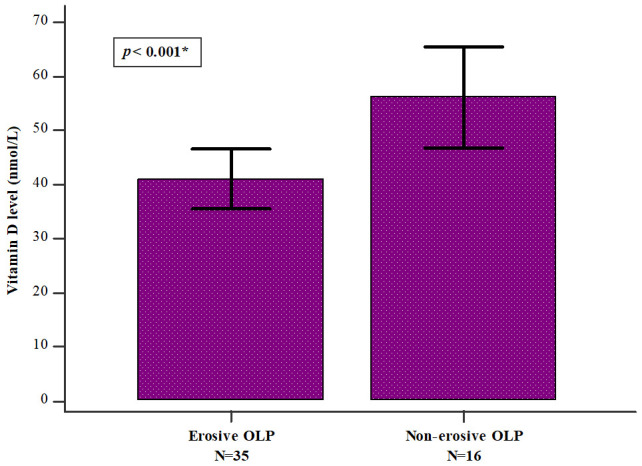
Comparison of serum vitamin D levels between the erosive and non-erosive OLP form groups. All variables are presented as mean ± standard deviation. * Student *t*-test.

Serum vitamin D levels were also lower in the erosive subgroup than in the non-erosive subgroup (40.4 ± 16.07 vs. 55.1 ± 17.1 nmol/L, *p* < 0.001) (Figure 4). The control mean (53.4 nmol/L) and the non-erosive OLP mean (55.1 nmol/L) both fell within the insufficiency range defined above.

## 4. Discussion

The main observation of this study was that circulating CST was higher in OLP patients than in controls and that CST was closely related to REU-based disease activity. The association was particularly evident in erosive OLP, where CST concentrations were markedly higher than in the non-erosive subgroup. These findings suggest that CST is more likely to reflect clinically active or erosive mucosal inflammation than the mere presence of OLP, and they expand the concept of a neuroendocrine-immune interface in this disease.

Increased serum CST in OLP is best interpreted within the broader neuroimmune biology of chronic mucosal inflammation. CST is generated from CgA, which is co-released with catecholamines during physiological and psychological stress [19,20]. OLP lesions are dominated by activated CD4+ and CD8+ T cells, Th1/Th17 skewing, altered Treg activity, and cytokines such as TNF-alpha, IFN-gamma, IL-6 and IL-17 [21,22,23,24]. These local processes may be linked to the systemic CST pool through stress-related CgA release, inflammation-enhanced CgA processing, and CST-mediated modulation of macrophages and leukocyte trafficking. Experimental and clinical data indicate that CST can reduce TNF-alpha, IL-1beta and IL-6, attenuate adhesion molecule expression, and limit immune cell chemotaxis [10,15]. In this framework, higher CST could represent a compensatory counter-regulatory response to sustained mucosal inflammation. Similar increases have been reported in several inflammatory or immune-mediated conditions, including inflammatory bowel disease, rheumatoid arthritis, severe COVID-19 and cardiovascular inflammatory states [6,7,8,25], whereas reduced levels have been reported in some metabolic and hypertensive phenotypes [26,27]. Thus, CST dysregulation appears to depend on disease context, inflammatory burden and neuroendocrine activation rather than following a single direction across all disorders. To our knowledge, this is one of the first studies to report elevated serum CST in OLP.

The correlation between CST and the REU total score further supports a relationship with clinical activity. REU scoring captures reticulation/keratosis, erythema and ulceration across multiple oral sites and has been shown to correlate with pain [18,28]. As lesions become erythematous or ulcerated, epithelial barrier disruption, cytokine release and immune-cell recruitment intensify. Such conditions may enhance CgA processing and CST release from neuroendocrine and immune-cell compartments. The parallel correlations with VAS pain and VAS burning indicate that CST also tracks patient-reported symptoms, although the overall median VAS scores were low (1 for pain and 2 for burning). These low values may reflect the inclusion of patients with limited or chronic lesions and confirm that symptom intensity should be interpreted together with objective REU scores rather than in isolation. Previous OLP biomarker studies have shown that angiopoietin-2, immunoglobulins, vitamin D and cortisol vary with disease severity [16]. Our findings add CST to this biomarker landscape and suggest a neuroendocrine dimension of OLP activity.

The higher CST concentrations observed in erosive OLP provide the clearest clinical signal in this dataset. Erosive OLP is considered a more severe phenotype, characterized by epithelial breakdown, greater symptom burden, CD8+ cytotoxic T-cell infiltration, TNF-alpha activity and basement membrane damage [29,30]. In contrast, non-erosive patterns usually show a more preserved epithelial barrier [31,32]. Therefore, the similarity between control and non-erosive CST medians in the present study is important: it suggests that CST may not be a general diagnostic marker for all OLP, but rather a marker of inflammatory escalation, ulceration or erosive activity. This interpretation is consistent with reports that vascular and humoral biomarkers, such as angiopoietin-2 and immunoglobulins, are higher in erosive disease [16]. However, because the non-erosive subgroup was small, this finding requires confirmation in a study powered for direct comparisons among controls, non-erosive OLP and erosive OLP.

Vitamin D was lower in the overall OLP group than in controls, in line with previous studies and meta-analytic data [33,34,35,36,37]. The mean vitamin D values in both controls and the non-erosive subgroup were within the insufficiency range, which helps explain why the control mean (53.4 nmol/L) was close to the non-erosive mean (55.1 nmol/L). In contrast, the erosive subgroup had lower levels, supporting a possible link between vitamin D status and more severe mucosal disease. Vitamin D signaling through VDR influences keratinocytes, dendritic cells, macrophages and T cells, promotes Treg differentiation and constrains Th1/Th17 responses [38,39]. Reduced vitamin D/VDR activity could therefore contribute to persistence of the inflammatory loop in OLP [35,40,41]. The lack of correlation between CST and vitamin D suggests that these biomarkers may reflect different aspects of disease biology: CST may be more closely related to neuroendocrine and inflammatory activation, whereas vitamin D reflects a VDR-centered immunoregulatory axis. Future studies should test whether vitamin D repletion changes CST levels or whether both markers add complementary information on disease activity.

Several limitations should be acknowledged. The cross-sectional design prevents causal inference, and residual confounding cannot be excluded. The study was single-center and the sample size, particularly for the non-erosive subgroup, was modest. Importantly, OLP is a localized mucosal disease, whereas CST was measured only in serum; salivary CST, lesional CgA/CST expression, immunohistochemistry and tissue transcriptomic analyses were not performed. Clinical grading was performed by one examiner and formal inter- and intra-examiner reliability coefficients were not available, which limits assessment of scoring reproducibility. Finally, although active corticosteroid therapy, antihypertensive treatment and vitamin D supplementation were exclusion criteria, detailed prior medication washout intervals and vitamin D category counts were not available, so residual medication- or nutritional-status confounding remains possible.

## 5. Conclusions

In this study, serum CST concentrations were increased in patients with OLP and correlated with REU total score, pain and burning. The most clinically informative finding was the higher CST concentration in erosive OLP, whereas non-erosive OLP showed CST values closer to controls. These results suggest that CST may be involved in the neuroendocrine-immune profile of OLP and may be more relevant as a marker of erosive or clinically active disease than as a general diagnostic marker for all OLP. Confirmation in larger prospective cohorts, ideally with salivary measurements and tissue-level CST/CgA expression, is required before CST can be proposed for routine disease monitoring.

## Figures and Tables

**Table 1:** Comparison of sociodemographic data and habits between the study groups.

Variable	Study Sample (*N* = 111)	Control Group (*N* = 60)	OLP Group (*N* = 51)	*p*
Age (years)	63.0 (56.0–68.7)	63.0 (57.0–66.5)	65.0 (53.0–71.7)	0.331 *
BMI (kg/m^2^)	23.6 ± 2.4	23.4 ± 2.3	23.8 ± 2.5	0.479 *
Sex (*N*, %)				
Male	34 (30.6)	22 (36.7)	12 (23.5)	0.197 ^†^
Female	77 (69.4)	38 (63.3)	39 (76.5)	
Smoking (*N*, %)	24 (21.6)	12 (20.0)	12 (23.5)	0.826 *
Every day alcohol consumption (*N*, %)	2 (1.8)	0 (0)	2 (3.9)	0.405 *

All variables are presented as whole numbers (percentage), mean ± standard deviation or median (interquartile range). * Mann–Whitney U test. ^†^ Chi-square test or Fisher’s exact test.

**Table 2:** Clinical data of the OLP group.

Variable	OLP Group (*N* = 51)
Type of OLP * (*N*, %)	
Reticular	47 (92.2)
Papular	1 (2.0)
Plaque-like	10 (19.6)
Atrophic	1 (2.0)
Erosive/ulcerative	35 (68.6)
Bullous	1 (2.0)
Exhibiting erosive/non-erosive subtypes (*N*, %)	
Non-erosive	16 (31.4)
Erosive	35 (68.6)
Localization * (*N*, %)	
Buccal mucosa	47 (92.2)
Gingiva	10 (19.6)
Dorsal tongue	7 (13.7)
Lateral tongue	6 (11.8)
Ventral tongue	7 (13.7)
Palate	5 (9.8)
Labial vestibulum	5 (9.8)
Oral floor	3 (5.9)
REU-R score	2.0 (2.0–3.0)
REU-E score	2.0 (0.0–5.7)
REU-U score	2.0 (0–4.0)
REU total score	6.0 (3.0–11.5)
VAS pain	1.0 (0–4.0)
VAS burning	2.0 (0–5.0)

All variables are presented as whole numbers (percentage) or median (interquartile range). * most patients had multiple types and locations.

**Table 3:** Serum catestatin levels correlation with clinical and demographic data in the OLP group (*N* = 51).

Variable	r	*p* *
Age (years)	−0.036	0.715
BMI (kg/m^2^)	0.007	0.942
REU-R score	0.265	0.078
REU-E score	0.748	<0.001
REU-U score	0.770	<0.001
REU total score	0.781	<0.001
VAS pain	0.708	<0.001
VAS burning	0.729	<0.001
Vitamin D (nmol/L)	−0.050	0.610

* Spearman’s correlation coefficient.

## Data Availability

The original contributions presented in this study are included in the article. Further inquiries can be directed to the corresponding author.
